# The adaptation of sport assessment-patella questionnaire into simplified Chinese version: cross-cultural adaptation, reliability and validity

**DOI:** 10.1186/s12955-020-01525-7

**Published:** 2020-08-05

**Authors:** Weizong Weng, Xin Zhi, Zhenyu Jia, Shanshan Liu, Jianming Huang, Fang Wan, Jia He, Shiyi Chen, Jin Cui

**Affiliations:** 1 Department of Orthopedics, Changhai hospital affiliated to the Naval Medical University, Shanghai, P. R. China; 2 Department of Orthopedics, Chenggong Hospital affiliated to Xiamen University, Xiamen, P. R. China; 3 Department of Health Statistics, the Second Military Medical University, Shanghai, China; 4 Department of Orthopedics, General Hospital of Southern Theater Command, Guangzhou, P. R. China; 5 Department of Sports Medicine, Huashan Hospital, No 12, Wulumuqi Zhong Road, Shanghai, 200040 P. R. China

**Keywords:** Patellar, Tendinopathy, VISA-P, Cross-cultural comparison, Chinese, Adaption, Validation study

## Abstract

**Background:**

The original version of Victorian Institute of Sport Assessment-Patella Questionnaire (VISA-P) is developed in English, and aimed to assess the severity of patellar tendinopathy symptoms. Before used in China, it should be translated to Chinese version.

**Objectives:**

Our aim is to make a translation/cross-culturally adaption for the VISA-P into simplified Chinese version (VISA-PC). And primarily validate the VISA-PC in Chinese speaking population.

**Methods:**

The translation process of VISA-P questionnaire into simplified Chinese version (VISP-PC) followed the International recognized guideline. Cross-cultural adaptation was carried out with a clinical measurement study. A total of 128 projects which consisted 33 healthy students, 39 patients with patellar tendinopathy and 56 military students (receive military training as at-risk population) were included into this study. Internal consistency was evaluated with Cronbach’s alpha, and test-retest reliability was assessed with intraclass correlation coefficients (ICCs). Construct validity and floor and ceiling effects were also tested.

**Results:**

The scores were 95.84 ± 5.97 of healthy group, 91.87 ± 9.03 of at-risk group, 62.49 ± 11.39 of pathological group. There is no ceiling and floor effect of VISA-PC. The Cronbach’s alpha (0.895) and ICC (0.986) values showed good internal consistency and reliability. There were high correlations between VISA-PC and Kujala patellofemoral score (*r* = 0.721). VISA-PC score also had good correlation with the relevant SF-36 items.

**Conclusion:**

The VISA-PC was well translated into simplified Chinese version (VISA-PC), which is reliable and valid for Chinese-speaking patients with patellar tendinopathy.

**Level of evidence:**

II.

## Introduction

Patellar tendinopathy is the most common overuse injury of the knee [12]. The major cause of the injury is repetitive and continuous stress on patellar and quadriceps [29]. The involved populations are usually athletes, especially basketball players, track and field athletes, and volleyball players [13, 15, 20, 29]. The participation of patients with tendinopathy in sports activities is restricted or even hindered [17].

The tendinopathy is a kind of non-inflammatory injury, which causes fibrotic scarring and collagen degeneration of the tendon [13]. The present diagnosis instruments are physical examination and ultrasound, and magnetic resonance imaging (MRI) is another. However, there is poor correlation between clinical symptoms and imaging results [5, 14]. Valid and reliable tools are needed to evaluate symptoms severity, physical function, and treatment effects.

The VISA-P was originally developed by an Austria institution, namely the Victorian Institute of Sports Assessment, and was originally designed for patellar tendopathy patients to assess the severity of symptom [27]. The VISA-P is a self-administered questionnaire originally constructed in English language. Since the first publication of VISA-P, it has also been applied to assess treatment responses, and has been adapted for various populations, including: Germany, Spanish, Korean, Greek, Italian, Dutch, Brazil, Swedish, French, and Turkish [3, 8, 10, 11, 16, 21, 22, 25, 31, 33]. Yet, no reliable and valid version of VISA-P into simplified Chinese has been adapted.

The purpose of this study is to conduct a translation of the VISA-P questionnaire from English into simplified Chinese version, and further cross-culturally adapt and validate it. It was hypothesized that the adaptation version of VISA-P into simplified Chinese (VISA-PC) as an instrument for patellar tendinopathy assessment would be reliable and valid in China.

## Materials and methods

### Translation and cross-cultural adaptation

**Fig. 1:**
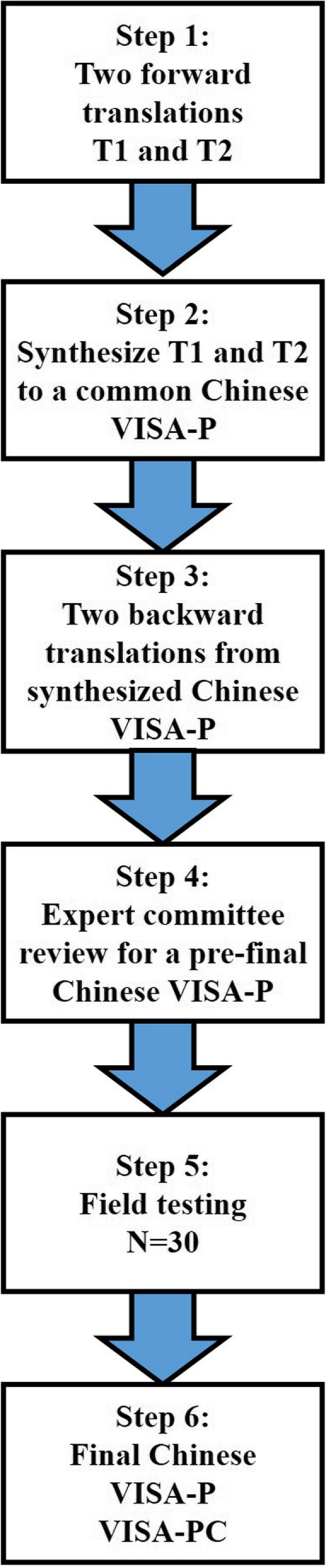
Flow chart of translation and cross-cultural adaptation of the Victorian Institute of Sport Assessment-Patella Questionnaire (VISA-P) to Chinese language

The whole process of translation and cross-cultural adaptation of the VISA-P followed the guideline and recommendations of the American Academy of Orthopedic Surgeons (AAOS) outcome committee [2]. The VISA was originally published in English [30]. The translation process was divided into five phases including: Phase No. 1, two independent translators including an orthopedic surgeon and a full-time translator were arranged to translate VISA-P into Chinese independently. Phase No. 2, two independent versions were merged to a common Chinese version, after reviewed by an expert committee and under the approval of the two translators. Phase No. 3, two bilingual English native speakers translated the merged VISA-P in Chinese back to English, which was aimed to take out conceptual errors. Phase No.4, a pre-final version of Chinese VISA-P was integrated with the cooperation between the four translators and the expert committee. Phase No. 5, the pre-final version was tested on a group of 30 people to make the final version understandable for native Chinese speakers. The flow chart of the translation and adaptation process was shown in Fig. 1.

### Participants and data collection

All the participates are from the Second Military Medical University. The inclusion criteria were as follows: 18 years old or older, can read Simplified Chinese and speak Chinese Mandarin. Before the inclusion, the participants were informed the basic information of this research, including the purpose and the detailed procedure, then signed their informed consents. This study included participants with different ethnics, educational, and social backgrounds.

Our study was approved by the clinical research ethics committee of Changhai hospital, the date of approval was 2016-03-01, and the protocol number was No.CH20160912.

There were respective inclusion criteria for the participants included in healthy group, at-risk group and pathological group. This study recruited 3 groups of projects: healthy group (33 healthy medical students), at-risk group (56 medical cadets who took military training involving jumping and running every day), and pathological group (39 students with patellar tendinopathy which was diagnosed with ultrasound examination). Healthy medical students and military medical cadets showed no symptoms of patellar tendinopathy, and students of pathological groups showed patellar tendinopathy symptoms.

All of the included participants completed the VISA-PC questionnaire twice. At the first time, the participants also completed the Short Form (36) Health Survey (SF-36), and Kujala patellofemoral score (Kujala scale) at the same time. The second-time of data collection of VISA-PC was finished 1 week after the first time for the assessment of test-retest reliability.

### Instruments

The instruments used in the present study included the Simplified-Chinese version of VISA-P (VISA-PC), and the Chinese version of the SF-36 and Kujala scale, which had been cross-culturally adapted and validated.

The VISA-P scale is the only specific scale for patellar tendinopathy, which consists of 8 items. The first 6 items were used to test pain during daily activities, and the other 2 items were used to represent the sports participation conditions. The maximum score of the first 7 items was 10 points, and of the last item was 30 points. The maximum 100 points of VISA-P indicate no symptom and fully functional. The minimum score of VISA-P theoretically was 0 points [30].

The SF-36 questionnaire is a questionnaire for assessing the patients’ general health status, which consists of 36 questions [32], with eight health concept subscales including, physical functioning (PF), bodily pain (BP), general health (GH), vitality (VT), social functioning [9], role-physical (RP), role-emotional (RE) and mental health (MH). The translated and cross-culturally adapted Chinese version of SF-36 questionnaire had already been introduced for clinical use [19].

The Kujala score consists of 13 items, it reflects the pain-related functioning and activities [18]. For Kujala scale, the maximum score is 100 points, which indicates full function and no pain-related symptom. The minimum score of Kujala score is 0 points.

### Psychometric assessments and statistical analysis

The statistical analysis was performed using SPSS for windows Release 22 (Chicago, IL). A *p* value of less than 0.05 was considered statistically significant. The sample size was in accordance with the principle of health status questionnaire study [27].

#### Ceiling and floor effects

The purpose of assessing the ceiling and floor effects was to observe and analyse the scores distribution. If the lowest score or highest score of the total questionnaire was greater than 15%, then the ceiling and floor effects were considered to occur [27].

#### Reliability

The reliability of VISA-P was estimated with two parameters, namely test-retest reliability and internal consistency. Internal consistency was assessed with Cronbach’s alpha, and the coefficient was also calculated for elimination of 1 item in all 8 questions. The value of the Cronbach’s alpha between 0.70 ~ 0.80 represents acceptable and the value between 0.80 ~ 0.90 represents good internal consistency [27]. Correlations between overall score and all the items were examined [26, 28].

The test-retest reliability was assessed with intraclass correlation coefficient (ICC) and the Bland-Altman plot. The result of ICC evaluation was divided into 5 categories, including excellent (*r* > 0.8), good (*r* = 0.61–0.80), moderate (*r* = 0.41–0.60), fair (*r* = 0.21–0.40) and poor (*r* ≤ 0.20) [1]. Bland-Altman plot was used to measure within-subject variation and limits of agreement [24].

#### Validity

Construct validity was evaluated with known group validity and concurrent validity.

Known group validity measures differences between the scores of different groups, which are healthy group, at-risk group, and pathological group. According to the former studies which refers to the VISA-P questionnaire, we expected significant differences between VISA-PC scores of pathological group and asymptomatic groups including healthy group and at-risk group, and there was no significant difference between VISA-PC scores of healthy group and at-risk group [8, 20, 30, 33]. In addition, the VISA-P scores of original VISA-P publication [30] and other adaptation studies (e.g. French, Germany, Turkish, Switish, Spanish, Italian, Dutch, Brazilian Portuguese and Korean versions) were also used to evaluate the VISA-PC validity [3, 8, 10, 11, 16, 21, 22, 25, 31, 33]. Here, we applied one-way analysis of variance (ANOVA) performed by SPSS 22.0.

Concurrent validity of VISA-PC was also evaluated, which was represented by the Pearson’s correlation coefficient (r) of VISA-PC with the Kujala score and SF-36 of Chinese adapted version. Correlations were categorized as follows: poor (*r* = 0–0.20), fair (*r* = 0.21–0.40), moderate (*r* = 0.41–0.60), very good (*r* = 0.61–0.80), or excellent (*r* = 0.8–1.0) [7]. We expected strong correlation of VISA-PC with Kujala scale, and PF and BP subscales of SF-36, moderate correlation with GH and RP subscales of SF-36, and poor correlation with mental health and social function, and vitality related subscales of SF-36, including SF, VT, RE, and MH subscales.

## Results

### Translation and cultural adaptation

No major problems were revealed during the forward and back-translations of VISA-P. Neither major problems during the cross-cultural adaptation. For better comprehension convenience of native Chinese-speaking population, some routine Chinese expressions were used to resolve linguistic discrepancies. The expert committee approved the final VISA-PC version.

### Descriptive statistics

Altogether 128 subjects, which were divided into healthy group (33), at-risk group (56), and pathological group (39), participated in in this study (Table S1). All the subjects are students of SMMU, the mean age of which was 20.7 ± 2.68, 21.4 ± 1.90, and 23.4 ± 1.40 years old, respectively. The pathological group indicated a significant elder age (*p*<0.0001), which implied that the elder population are more vulnerable to patellar tendinopathy. Among all the three groups, the gender ratio (male to female) was approximately 1.0.

All the 128 subjects finished the VISA-PC questionnaire, SF-36, and Kujala questionnaire at the beginning of the study for the 1-st test, and the VISA-PC scores were shown in Table 1. One week later, the 128 subjects finished the questionnaire for the 2rd-test to calculate the test-retest reliability (ICC), which is shown in Table 2.

**Table 1:** Results for each item of the VISA-PC (Mean ± SD)

Item	Healthy Group	At-risk Group	Pathological Group	Total
1	9.69 ± 0.52	9.48 ± 0.70	6.61 ± 1.02	8.66 ± 1.57
2	9.52 ± 0.70	9.55 ± 0.53	7.02 ± 1.49	8.77 ± 1.51
3	9.45 ± 0.74	9.45 ± 0.77	7.10 ± 1.17	8.73 ± 1.41
4	9.69 ± 0.52	8.71 ± 0.72	7.02 ± 1.44	8.45 ± 1.41
5	9.42 ± 0.49	9.17 ± 0.78	6.00 ± 1.41	8.27 ± 1.79
6	9.33 ± 0.63	9.30 ± 0.56	5.85 ± 1.58	8.25 ± 1.89
7	9.54 ± 1.07	9.19 ± 1.32	5.62 ± 1.64	8.19 ± 2.20
8	29.18 ± 2.59	27 ± 4.88	17.26 ± 4.37	24.59 ± 6.53
Total	95.84 ± 5.97	91.87 ± 9.03	62.49 ± 11.39	83.95 ± 17.06

*VISA-PC* Chinese version of the Victorian Institute of Sport Assessment-Patella, *SD* Standard deviation

**Table 2:** Test-retest reliability and ceiling/floor effects of the VISA-PC

	1st-Test (mean ± SD)	2nd-Test (mean ± SD)	ICC (95%CI)	Observed range	Floor effect (%)^a^	Ceiling effect (%)^a^
Healthy Group	95.84 ± 5.97 ^b^	95.81 ± 5.74	0.994 (0.960–0.998)	78–100	0.00	9.09
At-risk Group	91.87 ± 9.03 ^b^	91.68 ± 8.99	0.992 (0.982–0.999)	68–99	0.00	0.00
Pathological Group	62.49 ± 11.39	62.41 ± 11.12	0.999 (0.998–0.999)	44–88	0.00	0.00
Total	83.95 ± 17.06	83.83 ± 16.96	0.999 (0.998–0.999)	44–100	0.00	2.34

The 1st-Test was conducted at the beginning of this research (112 patients), the 2nd-Test was conducted one week later to calculate the test-retest reliability (ICC) of the VISA-PC (112 patients)

*ICC* intra-class correlation coefficient, *95% CI* 95% confidence interval, *VISA-PC* Chinese version of Victorian Institute of Sport Assessment-Patellar Tendinosis questionnaire

^a^ Percentage of patients with the worst (floor effect) and the best (ceiling effect) condition

^b^ Significant difference compared to Pathological Group

### Floor and ceiling effects

The VISA-PC scores showed good distribution, which ranged from 78 ~ 100 in healthy group, 68 ~ 99 in at-risk group, 44 ~ 88 in pathological group, and 44 ~ 100 in the whole sample. There were 3 subjects in healthy group who scored the highest score, and no subject who scored the minimum score, either in 1st test or 2rd test. There was no floor or ceiling effects of this scale, and no data was miss during the whole test.

### Reliability

The values of ICC (Table 2) and Cronbach’s alpha (Table S2) showed excellent reliability of VISA-PC.

**Fig. 2:**
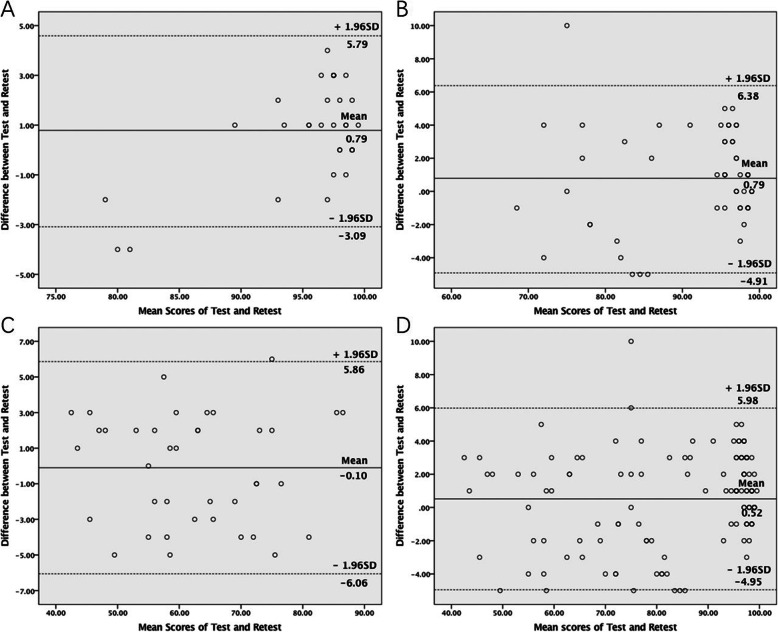
Bland-Altman plot showing differences between Test and Retest. **a** healthy group; **b** at-risk group; **c** pathological group; **d** the whole sample

There was no major difference between scores of VISA-PC in the 1st test and 2rd test after 1 week. The ICC was 0.986 (95%CI: 0.981 ~ 0.990) for the scores in the healthy group, 0.933 (95%CI: 0.869 ~ 0.966) for the scores in the at-risk group, 0.946 (95%CI: 0.910 ~ 0.968) for the scores in the pathological group, and 0.965 (95%CI: 0.934 ~ 0.981) for the scores of whole study sample. The ICC results indicated excellent test-retest reliability of VISA-PC. The Bland-Altman plot (Fig. 2) represented the systematic bias when compared between test and retest evaluation, and no bias was observed in this study.

The Cronbach’s alpha value in this study for the total scale was 0.895, which indicated the good internal consistency of VISA-PC questionnaire. The correlation of each item with the total score was totally well, and with the elimination of each item of the 8items resulted in an alpha on less than 0.870 (Table S2).

### Validity

Known group validity was confirmed with the comparison among VISA-PC scores of three groups. There was significant difference among the 3 groups, and the post hoc test indicated that there was a significant decrease of the scores of pathological group (*P* < 0.05) compared to both healthy group and at-risk group. No significant difference was observed between scores of healthy group and at-risk group. Besides, the VISA-PC results were consistent with former studies of VISA questionnaire clinical measurement studies (Table 3) [3, 8, 10, 11, 16, 21, 22, 25, 31, 33].

**Table 3:** VISA-PC scores compared with original and other adapted version scores

Version	Healthy Group	At-risk Group	Pathological Group
Chinese version	96 ± 6 (*n* = 33)	92 ± 9 (*n* = 56)	62 ± 11 (*n* = 39)
Original version (English)	95 ± 8 (*n* = 26)	75 ± 17 (*n* = 100)	55 ± 12 (*n* = 14)
French version	99 ± 2 (*n* = 22)	86 ± 14 (*n* = 42)	53 ± 17 (*n* = 28)
Germany version	95 ± 6 (*n* = 52)	……	62 ± 13 (*n* = 23)
Turkish version	94 ± 9 (*n* = 29)	81 ± 4 (*n* = 24)	59 ± 12 (*n* = 34)
Swidish version	83 ± 13 (*n* = 17)	79 ± 24 (*n* = 17)	48 ± 20 (*n* = 17)
Spanish version	96 ± 2 (*n* = 40)	90 ± 9 (*n* = 40)	56 ± 13 (*n* = 40)
Italian version	……	……	44 (*n* = 25)
Dutch version	95 ± 9 (*n* = 18)	89 ± 11 (*n* = 15)	58 ± 19 (*n* = 20)
Brazilian Portuguese version	……	……	59 ± 18 (*n* = 52)
Korean version	93 ± 9 (*n* = 5)	……	68 ± 16 (*n* = 23)

*VISA-PC* Chinese version of the Victorian Institute of Sport Assessment-Patella, *SD* Standard deviation

Concurrent validity was demonstrated with the correlation between VISA-PC and Chinese version of SF-36 and Kujala scores (Table 4). Optimal correlations of the VISA-PC with Kujala score and PF and BP subscales of SF-36 were observed (*r* = 0.721, *P* < 0.001; *r* = 0.792, *P* < 0.001; *r* = 0.656, *P* < 0.001, respectively). And there were moderate correlations of VISA-PC with RP and GH subscales of SF-36 (*r* = 0.554, *P* < 0.001; *r* = 0.594, *P* < 0.001, respectively). The correlations of VISA-PC with SF, MH, VT, and RE subscales were fair or poor (*r* = 0.440, *P* < 0.001; *r* = 0.289, *P* < 0.001; *r* = 0.251, *P* > 0.05; *r* = 0.237, *P* > 0.05, respectively).

**Table 4:** Constructive validity of the VISA-PC

Correlation coefficient r	VISA-PC
Kujala patellofemoral score	
Total score	0.721**
SF-36 subscales	
Physical Function, PF	0.792**
Role Physical, RP	0.554**
Bodily Pain, BP	0.656**
General Health, GH	0.594**
Vitality, VT	0.251
Social Function, SF	0.440**
Role Emotional, RE	0.237
Mental Health, MH	0.289**

*CH-VISA-P* Chinese version of Victorian Institute of Sport Assessment-Patellar Tendinosis questionnaire, *SF-36* Short Form 36

**: *p*<0.001

## Discussion

In our present study, a simplified Chinese version of VISA-P questionnaire VISA-PC was translated, adapted, and validated. The psychometric properties of VISA-PC were assessed, and the VISA-PC questionnaire was proved to be reliable and valid, which indicated that the simplified Chinese version was a suitable instrument to evaluate the clinical status of patellar tendinopathy for Chinese-speaking population. No missing data and major problem should be reported when the cross-cultural adaptation and evaluation were performed, which indicated VISA-PC was well accepted. According to our present study, the VISA-PC as a ideal instrument could be feasible and suitable for Chinese population for patellar tendinopathy evaluation.

Different from the former instruments which assessed the general function and sports ability to reflect the conditions of patellar tendinopathy [30], VISA-P was the only disease-specific instrument to assess patellar tendinopathy symptoms that impact the function and capacity in sports engagement. Before this study, there was no translated and cross-cultural adapted version of VISA-P for Chinese-speaking population. Kujala scale is a well-documented questionnaire for patients with patellofemoral pain, and was translated and adapted into Chinses version [4]. And SF-36 also had the translated and adapted version in Chinese, which was commonly applied in general health status evaluation of patients [9]. In this study, we used the correlation of VISA-PC with Kujala scale and SF-36 to evaluate the constructive validity of VISA-PC.

The floor or ceiling effects assessment could predict whether there would be overestimation of agreement parameters [6]. There was no maximum (100 points) nor minimum (0 points) scores of pathological group subjects, and the total maximum and minimum scores were both less than 15% of the whole sample. Thus no floor or ceiling effect of VISA-PC was observed in this study. Before our study, the studies of French [11] and Turkish [3] for VISA-P adaptation reported no floor or ceiling effect, either.

The ICC (Table 2) was calculated for test-retest reliability assessment of VISA-PC. According to the former study, 2 days to 2 weeks were recommended intervals for test-retest evaluation [23]. The previous studies which translated and cross-culturally adapted VISA-P into their languages chose 30 min, 2 h, 24 h, and 1 week as test-retest intervals [3, 8, 10, 11, 16, 21, 22, 25, 31, 33]. However, it was demonstrated by the French version [11] that short interval might cause recall effect. In this study, 7 days were chosen to avoid memory-based response, and there were probably no changes in the patellar tendinopathy status. The total ICC (0.986, 95%CI: 0.981–0.990) indicated excellent test-retest reliability.

High value the Cronbach’s alpha (0.895) confirmed high level of internal consistency, which indicated that there was no redundant item of VISA-PC. And the stability of Cronbach’s alpha when each item was deleted indicated the high level of correlation and balance among each item.

As expected, VISA-PC scores of pathological groups were significantly lower than those of healthy group and at-risk group. And there were similar results in the known group validity evaluation (Table 3) [3, 8, 10, 11, 16, 21, 22, 25, 31, 33].

Spearman correlation coefficients of VISA-PC with Kujala scale and SF-36 subscales confirmed the construct validity. As VISA-P is a specific scale for patellar tendinopathy, it was hypothesized that there would be relative high correlations with Kujala scale, and certain relevant subscales of SF-36, including PF and BP. The Construct validity results were consistency with the previous studies of VISA-P cross-cultural adaptation versions [3, 8, 10, 11, 16, 21, 22, 25, 31, 33].

There are two limitations to be discussed of our study. First, this is a study only referred to a single-centre. As China is such a country of vast territory, where population is widespread, including many ethnic groups, therefore multi-centre study will be more valuable to confirm the characteristics of the translated questionnaire. Second, the responsiveness was not evaluated in our study, and future investigation were needed.

## Conclusion

The VISA-P was successfully translated and cross-culturally adapted into Chinese version VISA-PC, which was proven to be a reliable and valid instrument for Chinses-speaking population. And the VISA-PC can be recommended as a robust tool for assessing the impact of symptoms on function and capacity of sports engagement in Chinese-speaking patients with patellar tendinopathy.

## Supplementary information

**Additional file 1: Table S1.** Demographic characteristics of participants.

**Additional file 2: Table S2.** Internal consistency of VISA-PC.

## Data Availability

Data are available upon reasonable request.

## Abbreviations

VISA-P: The Victorian Institute of Sports Assessment- Patellar questionnaire
VISA-PC: Simplified Chinese version of VISA-P
AAOS: The American Academy of Orthopedic Surgeons
ICC: Intraclass correlation coefficient
SMMU: The Second Military Medical University
ANOVA: Analysis of variance
