# Supplementary material for: The adaptation of sport assessment-patella questionnaire into simplified Chinese version: cross-cultural adaptation, reliability and validity
Source: Health Qual Life Outcomes. 2020 Aug 5;18:269. doi: 10.1186/s12955-020-01525-7 (PMC7409401; doi:10.1186/s12955-020-01525-7)
Supplement: Supplementary file 1 — Additional file 1: Table S1. Demographic characteristics of participants. [file 12955_2020_1525_MOESM1_ESM.docx]

| Table S1. Demographic characteristics of participants | | | |  |
| --- | --- | --- | --- | --- |
|  | Healthy Group | At-risk Group | Pathological Group | *p* value |
| Total number of patients | 33 | 56 | 39 |  |
| Age (Year) |  |  |  |  |
| Mean ± SD | 20.7 ± 2.68 | 21.4 ± 1.90 | 23.4 ± 1.40 | ＜0.0001 |
| Gender |  |  |  |  |
| Male (%) | 17（52%） | 28（50%） | 20（51%） | 0.988 |
| Female (%) | 16（48%） | 28（50%） | 19（49%） | 0.988 |
| SD= standard deviation |  |  |  |  |
