# Supplementary material for: The adaptation of sport assessment-patella questionnaire into simplified Chinese version: cross-cultural adaptation, reliability and validity
Source: Health Qual Life Outcomes. 2020 Aug 5;18:269. doi: 10.1186/s12955-020-01525-7 (PMC7409401; doi:10.1186/s12955-020-01525-7)
Supplement: Supplementary file 2 — Additional file 2: Table S2. Internal consistency of VISA-PC. [file 12955_2020_1525_MOESM2_ESM.docx]

| Table S2. Internal consistency of VISA-PC | | | |
| --- | --- | --- | --- |
| Question | Mean ± SD | Corrected item-total correlation | Alpha if item removed |
|  | if item deleted |  |  |
| 1 | 75.28±15.53 | 0.971 | 0.874 |
| 2 | 75.17±15.76 | 0.869 | 0.881 |
| 3 | 75.21±15.87 | 0.850 | 0.884 |
| 4 | 75.49±15.87 | 0.851 | 0.884 |
| 5 | 75.67±15.35 | 0.950 | 0.871 |
| 6 | 75.68±15.29 | 0.940 | 0.870 |
| 7 | 75.75±14.97 | 0.951 | 0.864 |
| 8 | 59.35±11.02 | 0.953 | 0.970 |
| Total score | 83.94±17.05 | 1.000 | 0.895 |
| SD= standard deviation | | | |
